# Exposure Assessment of methyl mercury from consumption of fish and seafood in Peninsular Malaysia

**DOI:** 10.1007/s11356-021-17483-6

**Published:** 2021-11-26

**Authors:** Nurul Izzah Ahmad, Wan Rozita Wan Mahiyuddin, Wan Nurul Farah Wan Azmi, Ruzanaz Syafira Ruzman Azlee, Rafiza Shaharudin, Lokman Hakim Sulaiman

**Affiliations:** 1 Environmental Health Research Centre, Institute for Medical Research, National Institutes of Health, Ministry of Health Malaysia, No. 1, Jalan Setia Murni U13/52 Seksyen U13, Setia Alam, 40170 Shah Alam, Selangor Malaysia; 2 Cancer Research Centre, Institute for Medical Research, Hematology Unit, Ministry of Health Malaysia, Jalan Pahang, 50588 Kuala Lumpur, Malaysia; 3 Centre for Environment and Population Health, Institute for Research, Development and Innovation, International Medical University, No. 126, Jalan Jalil Perkasa 19, Bukit Jalil, 57000 Kuala Lumpur, Malaysia

**Keywords:** Methyl mercury, Fish, Seafood, Exposure assessment, Food safety, Malaysia

## Abstract

The concentration of meHg in freshwater fish and seafood was investigated, as well as the consumption patterns of fish and seafood by different demographic groups (age, ethnicity, gender). A potential alarm for human health hazards was also assessed, and the results were compared to the provisional tolerable weekly intakes (PTWIs) and the hazard quotient parameter (HQ). The results showed that meHg levels of 67 species ranged from 0.013 to 0.252 mg/kg of wet weight (WW) with significant differences between different fish and seafood groups (χ^2^_KW_ = 49.09; *p* < 0.001). Median concentrations of meHg in fish and seafood groups in descending orders are as follows: demersal fish (0.1006 mg/kg WW) > pelagic fish (0.0686 mg/kg WW) > freshwater fish 0.045 mg/kg WW) > cephalopods (0.0405 mg/kg WW) crustaceans (0.0356 mg/kg WW). The results revealed that older population (> 40 years old) consumed significantly (*p* = 0.000) more fish compared to younger generations and the elderly consumed the highest amounts of fish (104.0 ± 113.0 g/day). The adolescents (10–17 years old) consumed more than double of amount for both cephalopod and crustacean compared to the older populations (*p* < 0.05). Malay ethnic (96.1 ± 99.6 g/day) consumed significantly (*p* = 0.000) higher amounts of fish and seafood compared to other ethnicities, similar to male subjects (95.2 ± 102 g/day; *p* = 0.026) when compared to the female (86 ± 96.3 g/day). The estimated weekly intake (EWI) values showed results below 1.6 µg/kg BW/week, the tolerable levels recommended by the Joint FAO/WHO Expert Committee on Food Additives (JECFA) for all different demographic factors except for higher consumers at 75th percentile and above. Consumption of marine fish contributed to a higher value of PTWI to all different demographic groups (the estimated weekly intake (EWI) range: 0.2988–0.6893 µg/kg BW/week) but for the adolescents, where from the consumption of crustaceans (0.3488 µg/kg BW/week or 21.8% of PTWI) and cephalopods (0.504 µg/kg BW/week or 31.5% of PTWI). The results from this study also revealed the HQ value for overall consumption of fish and seafood by the adolescents and elderly exceeded one. This was contributed from the consumption of demersal fish and cephalopods, thus indicating the nonacceptable level of noncarcinogenic adverse health effects.

## Introduction

Fish and seafood are a good source of energy and proteins, as well as key nutrients like minerals and vitamins, and they have a lot of health benefits (Mehouel et al 2019; Barone et al 2015). It also contains eicosapentaenoic acid (EPA), docosahexaenoic acid (DHA), and long-chain omega-3 polyunsaturated fatty acids (n-3 PUFAs), which are vital unsaturated fatty acids linked to excellent health (Barone et al 2015; Larsen et al. 2011; McManus et al. 2011). Medical research has shown that eating meat substitutes, fat fish or lean fish, and fish oil mixed with vegetables can improve fat consumption quality, lower calorie intake, and prevent lifestyle diseases, all of which are linked to a variety of health benefits, including maintaining healthy human hearts, brains, joints, and immune systems (Larsen et al. 2011; McManus et al. 2011).

Fish consumption, on the other hand, is the most prevalent way for humans to be exposed to mercury, which is released into the environment by both natural and human sources (Mehouel et al 2019). Mercury (Hg) exists mainly in different forms, an elemental mercury (HgO), inorganic mercury (Hg^+^, Hg^2+^), and organic mercury (MeHg^+^, EtHg^+^ PhHg^+^, and so on) (Mehouel et al 2019). In fish and seafood, often found is an organic form of meHg as this compound is predominant (Mehouel et al 2019; Morgano et al. 2011; Burger 2009). It occurs at a high percentage, ranging between 95 and 97% of total mercury (THg) when the compound accumulates in fish tissue. Hg linked to aquatic sediments is transformed into organic form by microbial activities through methylation and enzymatic processing, and it then enters the aquatic food chain, where it reaches its maximum concentration in predatory fish (Mehouel et al 2019; Clarkson et al. 2003). MeHg can be concentrated in fish either directly in the water or through food chain components. The ingestion of fish contaminated with meHg has received considerable critical attention due to the adverse health outcomes of neurologic damage such as mental retardation, seizures, vision and hearing lost, delayed development, language disorder, and memory loss as well as renal damage, reproductive disorders, and damage in the cardiovascular system (Mehouel et al 2019; Andrew et al 2016; Barone et al 2015). MeHg could potentially be one of the risk factors for infertility (Hsi et al 2016).

There are concerns about Malaysians being exposed to excessive levels of mercury as a result of eating fish and shellfish. Our earlier survey of people of various ethnicities in this country, conducted in the years 2008 to 2009, revealed that Malaysians had the second highest daily fish intake behind Japan among Asian nations and placed fifth in the world (Ahmad et al 2016). The maximum permitted proportion of meHg in seafood was set at 0.5 mg/kg in Malaysian Food Regulation 1985 (Food Act 1983, (Act 281) and Regulations 2006) (International Law Book Services 2006), which is the same level as the one set by the joint FAO/WHO Expert Committee (FAO/WHO 2006). In view of the above, the FAO/WHO Expert Committee on Food Additives (JECFA) has established provisional tolerable weekly intake (PTWI) for meHg at 1.6 µg/Kg/BW/week. This is the safe level of intake or the maximum amount of a contaminant that a person is weekly exposed to over a lifetime without intolerable risk of health effects associated with the consumption of food (Wan Azmi et al 2019; Kuras et al 2017). To estimate the potential of health risk due to exposure to the contaminant, the United State Environmental Protection Agency (USEPA) created the reference dose (RfD), an estimated daily oral exposure of contaminant to the human population that is likely to be without considerable risk of harmful effects during a lifetime which is set at 0.0001 mg/kg/day for Hg (Wan Azmi et al 2019; USEPA 2000).

Many research on metal pollution in fish and seafood have been conducted in Malaysia (Wan Azmi et al 2019; Low et al 2015; Alina et al 2012; Mok et al 2012; Kamaruzaman et al 2011; Agusa et al 2005), and a number of published data on THg or meHg levels in fish and seafood are available (Anual et al 2018; Ahmad et al. 2015a, b; Hajeb et al 2009). Many investigations found varying amounts of THg or meHg in a small number of fish and shellfish species gathered from specific locations around Malaysia. The results demonstrated that THg or meHg concentrations were low when compared to Malaysian Standards or JECFA recommended values. Several investigations found that some species of fish and shellfish seized from Malaysian markets have high THg or meHg contents, potentially putting consumers at risk (Ahmad et al. 2015a, b; Hajeb et al 2009, 2010; Agusa et al. 2005). Among those species are fork-tailed threadfin bream and big eye scads (Agusa et al 2005), longtail tuna, mackerel (Hajeb et al 2009), bluespotted stingray, honeycomb stingray, and John’s snapper (Ahmad et al. 2015a). An earlier study also reported that 48% of marine fish had Hg levels higher than the guideline value especially among carnivorous feeding (Agusa et al 2005). In this country, risk assessments studies on mercury have looked at the sources/locations, at-risk populations, fish species and families/groups, seafood preparation, and risk assessment methodologies (Agusa et al 2005, 2007; Hajeb et al 2009; Hajeb and Jinap 2011; Low et al 2015; Anual et al. 2018; Wan Azmi et al 2019). The quantities of trace elements in fish gathered throughout Southeast Asia varied greatly, and the estimated daily intake (EDI) value was calculated higher due to the high consumption rate of seafood in Malaysia. The EDI for all specimens of fork-tailed threadfin bream and sharp-tooth jobfishes exceeded the guideline values and would indicate hazardous to the populations in this region (Agusa et al 2005, 2007). Anual et al. (2018) reported that 14% of seafood had medium to high mercury concentrations with EWI higher than those with the PTWI for a few species of bream, snapper, croaker, barramundi, and tuna. In addition, their study showed the EWI values ranging from 2.1 to 4.0 μg/kg bw in listed fish (Anual et al 2018). The average Malaysian population was exposed to 1182 g per week or 73.95% of the PTWI (Hajeb et al 2009), whereas the exposures for the fisherman families were as high as 2,332 g per week or 145.8% of the PTWI (Hajeb and Jinap 2011). Instead, the probabilistic estimation of reasonable exposure and noncarcinogenic risks associated with consumption of freshwater fish (red tilapia) at 95th percentile exposure revealed hazard quotient (HQ) and hazard index (HI) values below 0.2 and 1, respectively, indicating that long-term consumption of this fish is likely to have negative consequences for Malaysians (Low et al 2015).

When assessing the risk of Hg or meHg from fish and shellfish diet, data from various other nations included more complete characteristics. There have been studies conducted to support regulatory analysis that rely on quantitative fish consumption estimates based on representative populations survey (von Stackelberg et al 2017) or involved a specific or broad range of seafood species consumed by population (Mehouel et al 2019; Budiyanto et al 2019; Barone et al 2015; Al-Mughairi et al 2013), collected from specific or different locations (Bhupander and Mukherjee 2011; Uratno et al. 2018) or wild and farm species (Chouvelon et al 2009). There are also studies that involved vulnerable groups of pregnant women, children below 17 years old, women of child-bearing age, and other high-risk consumers (Stuchal et al 2019; You et al 2018; Kuras et al 2017; Juric et al 2017; Andrew et al 2016; Whyte et al 2009). Data from other sources are also reported for common fish eaten or fish parts or organs, for example, fish muscle, liver, gills, kidney, and others (Matos et al 2018; Zolfaghari 2018). There are also studies that estimate the risk of Hg contamination from seafood eating by analyzing biological materials like blood and hair (You et al 2018; Ouboter et al 2018; Kuras et al 2017; Juric et al 2017). Current publications revealed results of THQ values for children and adults higher than one (Rakib et al 2021; Okati et al 2021) from consumption of selected commercial fish and its products, and the exposure among the indigenous communities are even higher from 3- to 25-folds compared to the USEPA reference dose or up to 11 times of the FAO guideline (Vasconcellos et al 2021).

This study updates information on the concentration of meHg in freshwater fish and seafood, including marine fish, cephalopods, and crustaceans, obtained at random from both fish landing ports and wholesale wet markets across Peninsular Malaysia. The study also looked at the consumption patterns of fish and seafood among Peninsular Malaysians of various age groups, ethnicities, and genders. Fish and seafood consumption for higher consumers was calculated at the 75th percentile and above, and data were compared to the median consumption at the 50th percentile. A potential alarm for human health hazards was evaluated, and results were compared to the PTWIs and through the parameter of HQ.

## Methodology

### Fish consumption survey

Between February 2008 and May 2009, a household-based cross-sectional study was undertaken in Peninsular Malaysia, with data collected through face-to-face interviews using predesigned questionnaires. Based on the National Household Sampling Frame (NHSF), Department of Statistics, Malaysia, a sampling frame made up of enumeration blocks (EBs) constructed for the 2000 Population and Housing Census was used to pick study subjects’ household addresses (Department of Statistics Malaysia 2000). The sample size was calculated using data from a Selangor population consumption survey, which revealed that 16.2% of the adult population consumed fish (153 g/person/day vs 944 g/person/day total food) (Ahmad 2007). Factors from two separate places (urban and rural), three different ethnic groups (Malay, Chinese, and Indian), and two different age groups were used. A number of 2,496 subjects were required in order to obtain a 95% confidence interval and 5% margin of error. Taking into account a 20% dropped-off rate, 2,996 subjects were identified from 1,500 household addresses received from the NHSF. In this study, a minimum of two adults and all adolescence ages 10 to 17 years were chosen from each family. The final count of 2,675 adults had completed the questionnaire. A number of 890 children/adolescents participated in the survey, but only 484 completed the questionnaires.

The questionnaires were divided into two sections. The first portion was a self-administered questionnaire that included sections on sociodemographic information, fish consumption patterns, frequency of fish intake, and knowledge, perceptions, and practices related to fish eating. The three copies of 24-h dietary diary forms made up the second half. In this portion, participants were instructed to keep track of what they ate and drank at each meal of the day. The interviewers were instructed how to read and understand the questionnaires and how to give instructions to the subjects. They were given a series of questionnaire tools which included pictures of serving dishes, fish commonly found in Malaysia, and common household measures like standard measuring cups, bowls, ladles, and spoons. The questionnaire was provided between the hours of 9:00 a.m. and 6:00 p.m.; however, interviewers were sometimes required to come at night if individuals were not at home during the day. Parents were asked to assist their children in filling out the 24-h dietary diary forms and answering questions on the surveys. Interviewers also rechecked all food recorded in dietary diary forms to verify the types and amount of food consumed by subjects.

The portion weight of food was determined using the “Atlas Makanan: Saiz pertukaran dan Porsi” (Suzana et al 2002, 2009) and “Nutrient and composition of Malaysian foods” (Tee et al 1997). If the food consumed was not listed in all of these references, the mean values were determined as the weight of that particular food from at least five different sources. The collections of the 3-day dietary diary were conducted during weekdays and weekends. Details on the calculation of sample size, questionnaires, and interviews involved adults from Peninsular Malaysia for the whole study entitled “Seafood consumption survey in Peninsular Malaysia, 2008–2009” were as described elsewhere (Ahmad et al 2016), while similar information for a survey that involved adolescence was reported recently (Ahmad et al 2019). Information on the demographic background of study subjects from both groups were presented in both published articles, accordingly (Ahmad et al 2016, 2019). The Medical Research and Ethics Committee (MREC) of the Ministry of Health Malaysia (MOH) approved the project, which was funded by the MOH. Informed consent and confidentiality were obtained from the subjects beforehand.

### Seafood collection and preparation

Sampling was conducted from June to December 2009. Samples were obtained from six major Fisheries Development Authority of Malaysia (LKIM) fish landing complexes and five wholesale wet marketplaces across Peninsular Malaysia. During three trips to each location, a total of 394 seafood samples were gathered. Details on how to collect, prepare, process, and store fish can be found elsewhere (Ahmad et al. 2015a, b).

### Determination of total mercury concentrations in seafood

In a microwave digestion device, a dried sample was digested (Multiwave 3000—Anton Paar). Total mercury was measured using a Perkin Elmer Flow Injection Mercury System (FIMS) apparatus with a programmable sample dispenser and a cold vapor atomic absorption spectrometry (AAS) methodology, as described by Mohd Fairulnizal et al. (1998). Analytical control was accompanied by the analysis of reagent blanks and standard reference samples. Details on the analysis were described elsewhere (Ahmad et al. 2015a, b). It was essential to convert mercury contents in fish samples to wet basis values, and the quantity of moisture content was determined using Tee et al. (1997) and other sources (Nurnadia et al. 2011). The results were compared to the recommended guideline levels by the joint FAO/WHO Expert Committee on Food Additives (FAO/WHO 2006) and the Malaysian Food Regulation 1985 (Food Act 1983, (Act 281), and Regulations 2006), under the Fourteenth Schedule of Regulation 38, the level at 0.5 mg/kg meHg in fish and seafood.

### Health risk assessment of mercury from seafood consumption

#### Estimated weekly intake (EWI) and maximum safe weekly consumption (MSWC)

The EWI of total Hg and/or meHg from consumption of seafood can be calculated by multiplying the total Hg and/or meHg contamination levels in seafood with the consumption levels per week dividing by the average body weight of the population by age group. The equation is as shown below:

$${EWI}=\frac{{Concentration of meHg }({mg}/{Kg WW}) \times {weekly consumption }({g})}{{Body weight }({kg})}$$

The typical adult body weight varies between 55 and 62 kg depending on demographic factors, while the average adolescent body weight is 45 kg (Ahmad et al 2016, 2019). The JECFA has defined a PTWI of 1.6 g/kg body weight/week for inorganic MeHg (WHO 2011).

MSWC to reach the PTWI for the Malaysian population at different sociodemographic characteristics was also estimated. The MSWC was calculated using the PTWI (g/kg body weight/week) value of 1.6 g/kg body weight/week multiplied by body weight for each population group and divided by total Hg and/or meHg concentrations:

$${PTWI at }1.6{ \mu g}/{kg body weight}/{week}=\frac{{Concentration of meHg }({mg}/{Kg WW}) \times {MSWC }({g})}{{Body weight }({kg})}$$

### Hazard quotient (HQ)

The risk assessment is a tool to estimate the probability of health effects due to exposure to the hazard, in which this study is the exposure through consumption of fish. The oral reference dosage (RfDs) for mercury was set at 1 × 10^–4^ (mg/kg-day) by the USEPA (Risk Information System (IRIS) (USEPA 2000). The HQ meHg was estimated using the equation below (Wan Azmi et al 2019):

$${HQ}=\frac{{EF}\times {ED}\times {FIR}\times {C}}{{RfD}\times {BW}\times { AT}}\times {10}^{-3}$$

where HQ is chemical-specific hazard quotient; EF is the exposure frequency (350 days/year); ED is the duration of human exposure for children and adults is 6 and 30 years, respectively; FIR is the seafood ingestion rate (based on total intake per day in gram by different groups of population); C is the metal concentration in the muscle of fishes (mg/kg wet weight); RfD is the oral reference dose (IRIS, USEPA); BW is the average body weight of population group (55 to 62 kg for adult, 45 kg for adolescent, and 60 kg for total population); and AT is the average time of human exposure to non-carcinogenic (ED × 365 days).

Target hazard is a ratio of the determined dose of a contaminant to an oral reference dose considered detrimental. HQ values were more than or equal to 1; it is assumed that the intake of meHg through the consumption of fish and seafood poses a potential noncarcinogenic health risk to the exposed population.

### Statistical analysis

IBM SPSS Statistics 26 was used to analyze the data. Before analyzing the THg data, it was cleaned and examined for discrepancies. The demographic features of different categories and groups were keyed in using data from a dietary survey. After completing the data entry, it was checked for any discrepancies, such as coding numbers, typo errors, and so on. At first, descriptive statistics were used to assess data normality using the one-sample Kolmogorov–Smirnov test, and/or the skewness of descriptive statistics was regulated between − 1 and + 1, whichever was true. Because of the outliers, the descriptive statistical analysis revealed that both groups of data were not regularly distributed. As a result, nonparametric statistics were employed. The medians and interquartile range were calculated. The Mann–Whitney U and Kruskall-Wallis tests were used to analyze differences across groups. A level of significance at 0.05 is set to determine the result is statistically significant. Significance values have been adjusted by the Bonferroni correction for multiple tests. Higher consumers were chosen among participants who consumed fish and seafood at the 75th, 90th, and 95th percentiles, and their consumption was compared to the median consumption at the 50th percentile.

## Results

### MeHg in fish and seafood

THg and meHg (median ± IQR) concentrations in marine and freshwater fish from fish landing ports and the wholesale markets in Peninsular Malaysia are summarized in Table 1. MeHg levels of 67 fish and seafood species ranged from 0.013 to 0.252 mg/kg of wet weight. Fish and seafood groups were comprised of 8 species of cephalopods, 12 species of crustaceans, 23 species of demersal fish, 1 species of freshwater fish, and 23 species of pelagic fish. Significant variations of meHg levels exist in different fish and seafood groups (χ^2^_KW_ = 137.486; *p* < 0.001). Among marine fish, the median for meHg levels was higher (> 0.1 mg/kg WW) in two species of pelagic fish (*Selar boops* and *Sarda orientalis*) and eight species of demersal fish (*Lutjanus argentimaculatus*, *Lutjanus russellii*, *Lates calcarifer*, *Psammoperca waigiensis*, *Dasyatis zugei*, *Nemipterus japonicus*, *Nemipterus furcosus*, and *Nemipterus nematophorus*). While for cephalopods and crustaceans as well as the freshwater catfish, meHg levels were nearly half compared to the marine fish at 0.045, 0.046, and 0.066 mg/kg WW, respectively. Pairwise comparisons using Bonferroni correction of meHg levels in fish and seafood groups showed significant differences between pelagic fish and the other three groups of demersal fish (*p* = 0.000); crustacean (*p* = 0.000), and cephalopod (*p* = 0.000). There were also significant differences between demersal fish and crustaceans (*p* = 0.000), cephalopod (*p* = 0.000), and freshwater fish (*p* = 0.048). Median concentrations of meHg in fish and seafood groups in descending order are as follows: demersal fish (0.1006 mg/kg WW) > pelagic fish (0.0686 mg/kg WW) > freshwater fish 0.045 mg/kg WW) > cephalopods (0.0405 mg/kg WW) crustaceans (0.0356 mg/kg WW). The calculation of median concentrations of meHg per species by means of the percentage to THg at 93%, 81%, and 50% for fish, cephalopods, and crustaceans, respectively (Anual et al 2018). These results showed that none of the meHg levels in fish and seafood groups exceeded both the national and international guidelines.

**Table 1:** Total Hg and meHg (mg/kg WW) levels in fish/seafood from the LKIM Complexes and wholesale market in Peninsular Malaysia

Common name	Species	*n*	total Hg (DW)	IQR	^#^MC (%)	Total Hg (WW)	*meHg (WW)
Pelagic fish							
Yellowstripe scad	*Selaroides leptolepis*	10	0.252	0.125	79.5	0.0517	0.0480
Oxeye scad	*Selar boops*	3	0.555	-	78.2	0.1210	0.1125
Bigeye scad	*Selar crumenopthalmus*	1	0.298	-	78.8	0.0632	0.0588
Yellowtail scad	*Atule mate*	4	0.458	0.304	76.8	0.1063	0.0988
Bigeye trevally	*Caranx sexfasciatus*	1	0.293	-	76.8	0.0680	0.0632
Greater amberjack	*Seriola dumerili*	1	0.203	-	84.7	0.0311	0.0289
Redtail scad	*Decapterus kurroides*	2	0.272	0.263	74.7	0.0688	0.0640
Round scad	*Decapterus muruadsi*	7	0.317	0.171	77.4	0.0716	0.0666
Slender scad	*Decapterus russelli*	4	0.195	0.108	74.7	0.0493	0.0459
Shortfin scad	*Decapterus macrosoma*	1	0.354	-	74.7	0.0896	0.0833
Torpedo scad	*Megalaspis cordyla*	17	0.319	0.198	74.8	0.0804	0.0748
Black pomfret	*Parastromateus niger*	8	0.242	0.121	76.5	0.0569	0.0529
Indian mackerel	*Rastrelliger kanagurta*	9	0.18	0.066	73.1	0.0484	0.0450
Faughn’s mackerel	*Rastrelliger faughni*	3	0.357	0.246	77.9	0.0789	0.0734
Indo-Pacific mackerel	*Rastrelliger brachysoma*	3	0.261	-	78.9	0.0551	0.0512
Slimmy mackerel	*Scomber australasicus*	10	0.269	0.065	77.7	0.0600	0.0558
Indo-Pacific king mackerel	*Scomberomorus guttatus*	9	0.262	0.355	75.9	0.0631	0.0587
Narrowbarred Spanish mackerel	*Scomberomorus commerson*	9	0.368	0.953	75.5	0.0902	0.0838
Dogtooth tuna	*Gymnosarda unicolor*	9	0.342	0.456	74.5	0.0872	0.0811
Striped bonito	*Sarda orientalis*	6	0.543	1.048	76.9	0.1254	0.1167
Longtail tuna	*Thunnus tonggol*	7	0.358	0.173	71	0.1038	0.0966
Frigate tuna	*Auxis thazard thazard*	2	0.237	-	76.8	0.0550	0.0511
Kawakawa	*Euthymus affinis*	2	0.289	-	75.2	0.0717	0.0667
	**Total**	**128**	**0.31**	**0.17**	**76.6**	**0.0738**	**0.0686**
**Demersal fish**							
Mangrove red snapper	*Lutjanus argentimaculatus*	3	0.856	-	75.8	0.2072	0.1927
Humpback red snapper	*Lutjanus gibbus*	1	0.436	-	82.1	0.0780	0.0726
Emperor red snapper	*Lutjanus sebae*	10	0.334	0.516	80.7	0.0645	0.0599
Malabar blood snapper	*Lutjanus malabaricus*	3	0.413	0.366	80.9	0.0789	0.0734
John's snapper	*Lutjanus russellii*	4	1.366	-	80.2	0.2705	0.2515
Giant sea perch	*Lates calcarifer*	7	0.537	0.436	78.1	0.1176	0.1094
Waigeu sea perch	*Psammoperca waigiensis*	4	0.532	0.165	79.1	0.1112	0.1034
Sharpnose stingray	*Himantura gerrardi*	8	0.384	0.741	79.1	0.0803	0.0746
Bluespotted stingray	*Neotrygon kuhlii*	6	0.492	1.251	82	0.0886	0.0824
Pale-edged stingray	*Dasyatis zugei*	4	0.548	0.509	76.1	0.1310	0.1218
Honeycomb stingray	*Himantura uarnak*	2	0.425	-	79.2	0.0884	0.0822
Reeve’s croaker	*Chrysochir aureus*	3	0.498	-	80.6	0.0966	0.0898
Tigertooth croaker	*Otolithoides ruber*	5	0.421	0.423	79.9	0.0846	0.0787
Soldier croaker	*Nibea soldado*	11	0.424	0.132	76.8	0.0984	0.0915
Bronze croaker	*Otolithoides biauritus*	1	0.069	-	79.9	0.0139	0.0129
Yellowbelly threadfin bream	*Nemipterus bathybius*	5	0.383	0.328	76.9	0.0885	0.0823
Japanese threadfin bream	*Nemipterus japonicus*	9	0.464	0.724	76.9	0.1072	0.0997
Forktail threadfin bream	*Nemipterus furcosus*	3	0.642	-	79.2	0.1335	0.1242
Threadfin bream	*Nemipterus thosaporni*	2	0.57	0.659	82.4	0.1003	0.0933
Fivelined threadfin bream	*Nemipterus tambuloides*	2	0.426	-	78.1	0.0933	0.0868
Doublewhip threadfin bream	*Nemipterus nematophorus*	2	1.211	-	80.4	0.2374	0.2207
Red filament threadfin bream	*Nemipterus marginatus*	2	0.244	-	76.2	0.0581	0.0540
Redspine threadfin bream	*Nemipterus nemurus*	1	0.298	-	79.5	0.0611	0.0568
	**Total**	**98**	**0.46**	**0.41**	**79.1**	**0.1082**	**0.1006**
	**Total marine fish**	**226**	**0.42**	**0.40**	**77.9**	**0.0910**	**0.0846**
**Freshwater fish**							
Catfish	*Clarias batrachus*	**9**	**0.334**	**0.325**	**77.1**	**0.0490**	**0.0450**
**Cephalopods**							
Golden cuttlefish	*Sephia esculenta*	6	0.257	0.11	81.4	0.0478	0.0387
Indian squid	*Sepia phuruonis*	10	0.199	0.16	81.4	0.0614	0.0497
Little squid	*Loligo duvaucelli*	4	0.249	-	81.4	0.0370	0.0300
Mitre squid	*Loligo uyii*	7	0.275	0.12	81.4	0.0463	0.0375
Old women octopus	*Loligo chinensis*	1	0.208	-	81.4	0.0512	0.0414
Pharoah cuttlefish	*Loligo sibogae*	2	0.33	-	81.4	0.0677	0.0548
Sibogae squid	*Loligo edulis*	6	0.364	0.51	81.4	0.0497	0.0402
Sword tip squid	*Cistopus indicus*	9	0.267	0.28	81.4	0.0387	0.0313
	**Total**	**45**	**0.25**	**0.13**	**81.4**	**0.0500**	**0.0405**
**Crustaceans**							
Banana prawn	*Penaeus merguiensis*	7	0.277	0.10	80.5	0.0540	0.0270
Giant tiger prawn	*Penaeus monodons*	2	0.399	-	80.5	0.0778	0.0389
Greasyback shrimp	*Penaeus semisulcatus*	3	0.251	-	80.5	0.0532	0.0266
Green tiger prawn	*Penaeus indicus*	2	0.273	-	80.5	0.0538	0.0269
Indian white prawn	*Penaeus japonicus*	8	0.276	0.13	80.5	0.2650	0.1325
Kuruma prawn	*Penaeus latisulcatus*	1	1.359	-	80.5	0.0710	0.0355
Pink shrimp	*Metapenaeus ensis*	4	0.28	0.50	80.5	0.0489	0.0245
Rainbow shrimp	*Metapenaeus affinis*	4	0.242	0.25	80.5	0.0546	0.0273
Sand velvet shrimp	*Parapenaeopsis sculptilis*	9	0.269	0.08	80.5	0.0472	0.0236
Spear shrimp	*Metapenaeopsis barbata*	3	0.176	-	80.5	0.0525	0.0262
Western king prawn	*Parapenaeospsis hardwickii*	5	0.364	0.17	80.5	0.0343	0.0172
Yellow shrimp	*Metapenaeus brevicornis*	4	0.215	0.05	80.5	0.0419	0.0210
	**Total**	**52**	**0.272**	**0.15**	**80.5**	**0.0712**	**0.0356**
	**Overall**	405	**0.06**	**0.05**	**78.74**	**0.0610**	**0.0305**

Total Hg in median ± IQR; DW, dry weight; IQR, interquartile range; MC, moisture content

^#^MC content was based on the works by Tee et al. (1997) and Nurnadia et al. (2011)

WW, wet weight; conversion of DW mercury concentrations in fish samples to WW were by means formula: DW = WW × (100/100-MC). Details on total mercury concentrations in seafood are referred to Ahmad et al. (2015a, b)

^*^Calculation of meHg concentrations were based on mean percentage of methylmercury to total mercury at 93% for fish, 81% for cephalopods and 50% for crustaceans (Anual et al 2018)

Comparison of meHg levels between different fish/seafood groups: χ^2^_KW_ = 49.090, *p* = 0.000, *N* = 405, Median = 0.061 ± 0.050 mg/kg WW

### Fish and seafood consumption at different demographic backgrounds

Fish and seafood consumption (g/day/person) (median ± IQR) by population in Peninsular Malaysia at different demographic categories were shown in Tables 2, 3, and 4. There are significant differences (*p* < 0.05) between the consumption of seafood among different age groups for all fish and seafood categories except for freshwater fish (Table 2). Overall, the results revealed that the older population (> 40 years old) consumed significantly (*p* = 0.000) more fish compared to younger generations (< 40 years old). The elderly consumed the highest amounts of fish (104.0 ± 113.0 g/day), but the difference is not significant when compared to the next age group of 41 to 60 years old (95.8 ± 99.8 g/day). Nevertheless, the differences are significant (*p* < 0.05) when compared to the other two age groups, the adolescents (10 to 17 years old) (84.9 ± 104.1 g/day), and the young adults (18 to 40 years old) (82.0 ± 89.1 g/day). In addition, the adolescents (10–17 years old) consumed about half amounts of marine fish (26.0 ± 30.6 g/day) compared to the older population, and the differences are significant at *p* = 0.000. Conversely, the amount they consumed were more than double for both cephalopod (80.0 ± 90.0 g/day) and crustacean (63.0 ± 65.0 g/day) compared to the consumption by the older populations (*p* < 0.05).

**Table 2:** Freshwater fish and seafood consumption (g/day/person) (median ± IQR) by population in Peninsular Malaysia at different age categories

Food category	Age by category				^#^*p*-value
	10–17 years (*n* = 653)	18–40 years (*n* = 1,209)	41–60 years (*n* = 1073)	≥ 61 years (*n* = 422)	
Pelagic fish	22.0 ± 32.7^a^	44.0 ± 60.7^b^	48.7 ± 66.4^bc^	48.3 ± 66.8^bc^	0.000
Demersal fish	30.5 ± 26.0^a^	46.0 ± 57.7^a^	35.0 ± 46.5^b^	52.0 ± 71.8^b^	0.017
Total marine fish	26.0 ± 30.6^a^	50.0 ± 77.0^b^	60.0 ± 77.8^bc^	66.0 ± 100.7^c^	0.000
Total freshwater fish	38.339.3	35.3 ± 45.4	36.7 ± 47.0	36.7 ± 77.3	0.790
Cephalopods	80.0 ± 90.0^a^	30.0 ± 37.0^b^	40.0 ± 31.9^bc^	29.8 ± 31.9^bc^	0.000
Crustaceans	63.0 ± 65.0^a^	13.3 ± 26.7^b^	21.0 ± 28.9^bc^	13.3 ± 22.9^bc^	0.000
Overall consumption	84.9 ± 104.1^a^	82.0 ± 89.1^a^	95.8 ± 99.8^b^	104.0 ± 113.0^bc^	0.000

Age categories:10–17 years, adolescents;18–40 years, young adults; 41–60 years, older adults; ≥ 61 years–elderly; IQR, interquartile range

^#^Kruskal–Wallis test were applied

Different alphabet within the different columns indicated significant differences (*p* < 0.05)

**Table 3:** Freshwater fish and seafood consumption (g/day/person) (median ± IQR) by different ethnicities in Peninsular Malaysia

Food category	Ethnicity			^#^*p*-value
	Malays (*n* = 2,592)	Chinese (*n* = 457)	Indians (*n* = 270)	
Pelagic fish	58.7 ± 64.0^a^	55.3 ± 46.5^a^	20.0 ± 79.0^c^	0.009
Demersal fish	46.0 ± 49.0	37.3 ± 53.2	42.3 ± 37.6	0.763
Total marine fish	69.5 ± 73.3^a^	45.0 ± 82.7^ab^	73.3b^c^	0.046
Total freshwater fish	36.7 ± 45.7	42.7 ± 30.8	44.0	0.532
Cephalopods	47.8 ± 42.0	53.3 ± 0.0	26.7 ± 28.3	0.741
Crustaceans	21.3 ± 20.7	3.8 ± 0.0	70.0 ± 36.5	0.908
Overall consumption	96.1 ± 99.6^a^	66.0 ± 88.0^b^	60.0 ± 64.5^bc^	0.000

IQR, interquartile range

^#^Kruskal–Wallis test was applied

Different alphabet within the different columns indicated significant differences (*p* < 0.05)

**Table 4:** Freshwater fish and seafood consumption (g/day/person) (median ± IQR) by different gender in Peninsular Malaysia

Food category	Gender			^#^p-value^1^	^#^p-value^2^
	Female (*n* = 1,859)	*Female (reproductive age) (*n* = 1,091)	Male (*n* = 1,495)		
Pelagic fish	70.7 ± 73.3	40.0 ± 60.7	58.7 ± 49.7	0.234	0.057
Demersal fish	50.0 ± 58.0	41.2 ± 50.2	47.5 ± 54.5	0.876	0.980
Total marine fish	58.7 ± 91.0	44.0 ± 76.8 ^a^	53.7 ± 75.7^b^	0.215	0.034
Total freshwater fish	35.3 ± 43.9	35.0 ± 35.7	46.0 ± 44.1	0.231	0.116
Cephalopods	43.3 ± 31.7	31.9 ± 34.1	64.5 ± 40.3	0.231	0.329
Crustaceans	26.7 ± 33.3	15.0 ± 33.2	29.3 ± 34.2	0.631	0.223
Overall consumption	86.0 ± 96.3^a^	81.0 ± 87.9^b^	95.2 ± 102^ab^	0.026	0.002

IQR, interquartile range

^*^Age between 15\ and 49 years old

^#^Kruskal–Wallis test was applied

*p* value^1^, differences between female and male

*p* value^2^, differences between female at selected reproductive age and male

Different alphabet within the different columns indicated significant differences (*p* < 0.05)

Fish and seafood consumption by three different major ethnicities in Peninsular Malaysia were shown in Table 3. The overall fish and seafood consumption was led by the Malay ethnic (96.1 ± 99.6 g/day) when compared to the Chinese (66.0 ± 88.0 g/day) and Indians (60.0 ± 64.5 g/day). The differences are significant at *p* = 0.000. The Chinese significantly (*p* = 0.046) consumed lesser marine fish compared to the other two ethnic groups. In the contrary, the Indians consumed significantly (*p* = 0.009) the least pelagic fish compared to the Malay ethnic and the Chinese. No significant differences (*p* > 0.05) were shown by different ethnicity towards consumption of demersal fish and freshwater fish, as well as cephalopods and crustaceans. Table 4 showed the consumption of different categories of fish and seafood by different genders. The overall results showed that fish and seafood consumption by male subjects (95.2 ± 102 g/day) were significantly (*p* = 0.026) higher when compared to the female (86 ± 96.3 g/day) and female at reproductive age (15 to 49 years old) (81 ± 87.9 g/day). But no significant differences (*p* > 0.05) were shown for the other food categories between different genders except for total marine fish. Female at reproductive age consumed the least (44 ± 76.85 g/day) of this fish categories compared to the other two groups. Table 5 showed consumption of fish and seafood by higher consumers. The consumption rates by the third quartile (75th percentile) consumers were 2.1 to 3.6 times greater than that of the median consumers (50th percentile). The rates were even higher for the 90th and 95th percentile consumer groups at 2.9 to 4.4 times and 3.7 to 5.3 times, respectively.

**Table 5:** Freshwater fish and seafood consumption (g/day/person) *(median ± IQR) by average and high consumers in Peninsular Malaysia

Food category		Higher consumer (percentiles)		
	50	75	90	95
Pelagic fish	58.3 ± 63.8	124.3 ± 53.8	171.0 ± 63.8	228.2 ± 51.0
Demersal fish	43.7 ± 48.7	105.0 ± 48.4	138.0 ± 92.0	200.1 ± 120.7
Total marine fish	51.1 ± 77.3	149.5 ± 75.6	199.0 ± 71.7	236.1 ± 67.9
Total freshwater fish	36.7 ± 45.7	104.0 ± 63.7	151.7 ± 58.6	193.5 ± 55.6
Cephalopods	45.0 ± 35.2	120.0 ± 67.0	148.3 ± 59.3	167.5 ± 50.7
Crustaceans	22.9 ± 30.2	64.0 ± 40.2	100.5 ± 50.0	108.2 ± 71.6
Overall consumption	89.0 ± 100.7	322.0 ± 101.9	380.5 ± 117.2	405.8 ± 110.7

IQR, interquartile range

### Health risk assessment (EWI, MSCW, and HQ)

Health risk assessment (EWI, MSCW, and HQ) of meHg from consumption of fish and seafood by populations in Peninsular Malaysia at different demographic factors were shown in Table 6, and the EWI were expressed in microgram per unit body weight per week (µg/kg BW/week). The EWI estimated values showed results below 1.6 µg/kg BW/week, the acceptable or tolerable levels recommended by the Joint FAO/WHO Expert Committee on Food Additives (JECFA) for all different demographic factors except for higher consumers at 75th percentile and above. The EWI for overall consumption of fish and seafood for higher consumers exceeded more than two times compared to the PTWI values, and major sources of meHg intake were from the consumption of both the pelagic and demersal fish. The EWI value for overall consumption of fish and seafood consumed by the 50th percentile consumers was 0.6443 µg/kg BW/week (Table 6), and this value is corresponding to 40% of the PTWI. Major sources of meHg were from the consumption of demersal (0.5149 µg/kg BW/week) and pelagic (0.4693 µg/kg BW/week) fish at 32% and 29% of the PTWI value, respectively. Consumption of marine fish contributed to a higher value of PTWI to all different demographic groups (the EWI range: 0.2988–0.6092 µg/kg BW/week) (Table 6) except for the adolescents, where the major sources for this group are from the consumption of crustaceans (0.3488 µg/kg BW/week or 22% of PTWI) and cephalopods (0.504 µg/kg BW/week or 32% of PTWI). These contributed to the EWI values of 0.8196 µg/kg BW/week or 51% of the PTWI for the overall consumption of fish and seafood among the adolescents. The value is at the highest rank when compared to the EWI values from the consumption of fish and seafood of the other groups.

**Table 6:** Health risk assessment (EWI, MSCW and HQ) of meHg from consumption of fish/seafood by populations in Peninsular Malaysia at different demographic factors

Demographic factors	*n*	BW (kg)	meHg (mg/kg) WW	Fish intake (g/day)	*EWI	% PTWI	^#^MSWC (kg)	HQ
Different age groups								
**Age 10–17 years old**	653							
Pelagic fish	135	45	0.069	22.0	0.2361	15	1.0435	0.3235
Demersal fish	28	45	0.101	30.5	0.4792	30	0.7129	0.6564
Total marine fish	156	45	0.074	26.0	0.2988	19	0.9745	0.4093
Total freshwater fish	33	45	0.045	38.3	0.2681	17	1.6000	0.3673
Cephalopods	43	45	0.041	80.0	0.5040	32	1.7778	0.6904
Crustaceans	46	45	0.036	63.0	0.3488	22	2.0227	0.4779
Overall consumption	505	45	0.062	84.9	0.8196	51	1.1602	1.1227
**Age 18–40 years old**	1,209							
Pelagic fish	546	60	0.069	44.0	0.3542	22	1.3913	0.4852
Demersal fish	95	60	0.101	46.0	0.5420	34	0.9505	0.7425
Total marine fish	581	60	0.074	50.0	0.4310	27	1.2993	0.5904
Total freshwater fish	53	60	0.045	35.3	0.1853	12	2.1333	0.2539
Cephalopods	71	60	0.041	30.0	0.1418	9	2.3704	0.1942
Crustaceans	104	60	0.036	13.3	0.0552	3	2.6970	0.0757
Overall consumption	894	60	0.062	82.0	0.5937	37	1.5470	0.8132
**Age 41–60 years old**	1,073							
Pelagic fish	562	65	0.069	48.7	0.3619	23	1.5072	0.4957
Demersal fish	118	65	0.101	35.0	0.3807	24	1.0297	0.5215
Total marine fish	602	65	0.074	60.0	0.4774	30	1.4076	0.6540
Total freshwater fish	61	65	0.045	36.7	0.1779	11	2.3111	0.2436
Cephalopods	80	65	0.041	40.0	0.1745	11	2.5679	0.2390
Crustaceans	93	65	0.036	21.0	0.0805	5	2.9217	0.1103
Overall consumption	193	65	0.062	95.8	0.6402	40	1.6759	0.8770
**Age ≥ 60 years old**	422							
Pelagic fish	209	59	0.069	48.3	0.3954	25	1.3681	0.5417
Demersal fish	60	59	0.101	52.0	0.6231	39	0.9347	0.8536
Total marine fish	223	59	0.074	66.0	0.5786	36	1.2777	0.7925
Total freshwater fish	15	59	0.045	36.7	0.1959	12	2.0978	0.2684
Cephalopods	18	59	0.041	29.8	0.1432	9	2.3309	0.1962
Crustaceans	27	59	0.036	13.3	0.0562	4	2.6520	0.0769
Overall consumption	340	59	0.062	104.0	0.7657	48	1.5212	1.0489
Different ethnicity								
**Malays**	1,495							
Pelagic fish	1,219	59	0.069	58.7	0.4805	30	1.3681	0.6583
Demersal fish	264	59	0.101	46.0	0.5512	34	0.9347	0.7551
Total marine fish	1,314	59	0.074	69.5	0.6092	38	1.2777	0.8346
Total freshwater fish	150	59	0.045	36.7	0.1959	12	2.0978	0.2684
Cephalopods	196	59	0.041	47.8	0.2297	14	2.3309	0.3146
Crustaceans	219	59	0.036	21.3	0.0900	6	2.6520	0.1232
Overall consumption	2,116	59	0.062	96.1	0.7075	44	1.5212	0.9692
**Chinese**	457							
Pelagic fish	125	60	0.069	55.3	0.4452	28	1.3913	0.6098
Demersal fish	21	60	0.101	37.3	0.4395	27	0.9505	0.6021
Total marine fish	131	60	0.074	45.0	0.3879	24	1.2993	0.5314
Total freshwater fish	5	60	0.045	42.7	0.2242	14	2.1333	0.3071
Cephalopods	10	60	0.041	53.3	0.2518	16	2.3704	0.3450
Crustaceans	15	60	0.036	3.8	0.0158	1	2.6970	0.0216
Overall consumption	274	60	0.062	66.0	0.4778	30	1.5470	0.6546
**Indians**	270							
Pelagic fish	89	55	0.069	20.0	0.1756	11	1.2754	0.2406
Demersal fish	10	55	0.101	42.3	0.5437	34	0.8713	0.7449
Total marine fish	95	55	0.074	73.3	0.6893	43	1.1911	0.9442
Total freshwater fish	1	55	0.045	44.0	0.2520	16	1.9556	0.3452
Cephalopods	6	55	0.041	26.7	0.1376	9	2.1728	0.1885
Crustaceans	36	55	0.036	70.0	0.3171	20	2.4722	0.4344
Overall consumption	196	55	0.062	60.0	0.4739	30	1.4181	0.6492
Different gender								
**Male**	1,495							
Pelagic fish	645	62	0.069	58.7	0.4573	29	1.4377	0.6264
Demersal fish	130	62	0.101	47.5	0.5417	34	0.9822	0.7420
Total marine fish	690	62	0.074	53.7	0.4480	28	1.3426	0.6136
Total freshwater fish	83	62	0.045	46.0	0.2337	15	2.2044	0.3202
Cephalopods	97	62	0.041	53.3	0.2437	15	2.4494	0.3339
Crustaceans	112	62	0.036	28.0	0.1125	7	2.7869	0.1541
Overall consumption	1,142	62	0.062	95.2	0.6670	42	1.5986	0.9137
**Female**	1,859							
Pelagic fish	806	57	0.069	70.7	0.5991	37	1.3217	0.8207
Demersal fish	171	57	0.101	50.0	0.6202	39	0.9030	0.8496
Total marine fish	871	57	0.074	58.7	0.5326	33	1.2344	0.7296
Total freshwater fish	79	57	0.045	35.3	0.1951	12	2.0267	0.2672
Cephalopods	115	57	0.041	43.3	0.2154	13	2.2519	0.2950
Crustaceans	158	57	0.036	26.7	0.1167	7	2.5621	0.1599
Overall consumption	1,474	57	0.062	86.0	0.6554	41	1.4696	0.8978
Female **(reproductive age)	1,091							
Pelagic fish	477	59	0.069	40.0	0.3275	20	1.3681	0.4486
Demersal fish	86	59	0.101	41.2	0.4937	31	0.9347	0.6763
Total marine fish	514	59	0.074	44.0	0.3857	24	1.2777	0.5284
Total freshwater fish	49	59	0.045	35.0	0.1869	12	2.0978	0.2560
Cephalopods	74	59	0.041	31.9	0.1533	10	2.3309	0.2100
Crustaceans	96	59	0.036	15.0	0.0633	4	2.6520	0.0868
Overall consumption	841	59	0.062	81.0	0.5964	37	1.5212	0.8169
Consumption rate by percentile								
**Median (50 percentile)**	3,357							
Pelagic fish	1452	60	0.069	58.3	0.4693	29	1.3913	0.6429
Demersal fish	301	60	0.101	43.7	0.5149	32	0.9505	0.7054
Total marine fish	1562	60	0.074	51.1	0.4405	28	1.2993	0.6034
Total freshwater fish	162	60	0.045	36.7	0.1927	12	2.1333	0.2639
Cephalopods	212	60	0.041	45.0	0.2126	13	2.3704	0.2913
Crustaceans	270	60	0.036	22.9	0.0951	6	2.6970	0.1303
Overall consumption	2,619	60	0.062	89.0	0.6443	40	1.5470	0.8827
High consumer								
**(75th percentile)**	657							
Pelagic fish	323	60	0.069	124.3	1.0006	63	NC	1.3707
Demersal fish	72	60	0.101	105.0	1.2373	77	NC	1.6949
Total marine fish	368	60	0.074	149.5	1.2887	81	NC	1.7653
Total freshwater fish	36	60	0.045	104.0	0.5460	34	NC	0.7479
Cephalopods	57	60	0.041	120.0	0.5670	35	NC	0.7767
Crustaceans	73	60	0.036	64.0	0.2658	17	NC	0.3641
Overall consumption	148	60	0.062	322.0	2.3312	146	NC	3.1935
**(90th percentile)**	264							
Pelagic fish	117	60	0.069	171.0	1.3766	86	NC	1.8857
Demersal fish	33	60	0.101	138.0	1.6261	102	NC	2.2275
Total marine fish	181	60	0.074	199.0	1.7153	107	NC	2.3498
Total freshwater fish	13	60	0.045	151.7	0.7964	50	NC	1.0910
Cephalopods	39	60	0.041	148.3	0.7007	44	NC	0.9599
Crustaceans	34	60	0.036	100.5	0.4174	26	NC	0.5717
Overall consumption	81	60	0.062	380.5	2.7548	172	NC	3.7737
**(95th percentile)**	131							
Pelagic fish	47	60	0.069	228.2	1.8370	115	NC	2.5165
Demersal fish	17	60	0.101	200.1	2.3578	147	NC	3.2299
Total marine fish	109	60	0.074	236.1	2.0351	127	NC	2.7879
Total freshwater fish	6	60	0.045	193.5	1.0159	63	NC	1.3916
Cephalopods	22	60	0.041	167.5	0.7914	49	NC	1.0842
Crustaceans	28	60	0.036	108.2	0.4493	28	NC	0.6155
Overall consumption	58	60	0.062	405.8	2.9379	184	NC	4.0246

BW, body weight (kg); WW, wet weight; *EWI, estimated weekly intake (µg/kg BW/week)

^**^age between 15 and 49 years old

Provisional tolerable weekly intake (PTWI) for MeHg = 1.6 μg/kg BW/week (WHO 2011)

MSWC, maximum safe weekly consumption (kg);

NC-MSCW for higher consumer (75th, 90th, 95th centile) was not calculated as the EWI values for all fish, and seafood groups were either nearly or above the PTWI

HQ, hazard quotient

MSWC values in kg are given on fish and seafood by group basis (Table 6). The amounts of demersal fish from Peninsular Malaysia which should be consumed by all population groups to reach the PTWI for meHg would be below 1 kg/week and very much lower (< 713 g/week) for the adolescents. The adolescents were allowed to consume < 2 kg per week (range: 1.04–2.02 kg/week) for all types of fish and seafood: to be more specific, about a kilogram for pelagic fish, one and a half kilogram to two kilograms of freshwater fish, the cephalopods, and crustaceans. While for the adults, they were allowed to consume a greater amount (< 2.5 kg/week) of marine and freshwater fish per week.

Table 6 also showed a summary of the HQ values for meHg from the consumption of fish and seafood by population in Peninsular Malaysia at different demographic backgrounds. The HQ is an integrated risk index that compares the ingested amount of meHg with the standard reference dose. The results from this study revealed that the overall consumption of fish and seafood by the adolescents and the elderly exceeded one. This is indicating the nonacceptable level of noncarcinogenic adverse health effects. Still, the HQ < 1 for the other groups of population at different types of fish and seafood categories assumes that daily exposure is not likely to cause negative health effects during the lifetime of the Malaysian population. High HQ values were contributed from the consumption of the demersal fish which exceeded a range between 0.5 and 0.8 or nearly 90% of the HQ values. For the adolescents, high HQ values were also contributed from the consumption of demersal fish, but the highest is from consumption of cephalopods. The high consumer at 75th percentile and above, the HQ values reached of up to 4.3 which indicated the presence of adverse effects due to meHg intoxication.

## Discussion

This study was carried out to assess the potential health risk of meHg exposure to population of Peninsular Malaysia from consumption of freshwater fish and seafood. We used two approaches for the estimation of human health risks to meHg in fish and seafood; the widely applied is the comparison with the PTWIs which representing the amount of meHg that can be ingested over a lifetime without appreciative risks (WHO 2011). Another approach is to determine risk through the value of HQ (USEPA 2000). This HQ value is an integrated index that compares the ingested amount of meHg with the standard reference dose where HQ < 1 signifies that the level of exposure is lower than the reference dose. It is assumed that a daily exposure at this level is not likely to cause any negative health effects during a lifetime in a human population.

It is well documented that meHg occurs at a high percentage in fish muscle and is the most toxic form of Hg. We analyzed THg in freshwater fish and seafood; these data were used to estimate meHg intakes for risk assessment data. In our earlier published data (Ahmad et al. 2015a, b), we presented and discussed in detail on THg levels in 46 species of commonly consumed marine fish samples and other seafood as well (cephalopods; 8 species and crustaceans; 12 species). The relationship between THg levels and size of samples (length and weight) was also discussed, and THg burden sampled from fish and seafood at different habitats, family group, and areas was compared. Previous results revealed only 1%, or three samples of demersal fish (bluespotted singray (*Neotrygon kuhlii*), honeycomb stingray (*Himantura uarnak*), and John’s snapper (*Lutjanus ruselli*)) had very high levels of THg (Ahmad et al 2015a). However, only one sample exceeded the Malaysian and international guidelines (FAO/WHO 2006; Malaysian Food Regulation 1985) when considering 95% or more of THg in the edible portion of seafood in the form of meHg (Khaniki et al 2005). The previous results for THg levels in crustaceans and cephalopods were either similar or reasonably low when compared to values published in various locations throughout the world (Ahmad et al 2015b), and none of these samples surpassed the guidelines. As data for meHg analysis in seafood for this country is scanty, we used levels recently reported by Anual and co-workers (Anual et al. 2018) for the nearest estimation, yet, only one sample each was analyzed for crustacean (*Metapenaeus affinis*) and cephalopod (*Loligo duvauceli*). The recalculated levels of meHg in fish and seafood were shown in Table 1. The results showed significant differences (*p* < 0.05) between pelagic fish and the other three groups of demersal fish, crustacean, and cephalopod (χ^2^_KW_ = 49.090, *p* = 0.000, *N* = 405, median = 0.061 ± 0.050 mg/kg WW). Concentrations of meHg in fish and seafood groups showed the highest in demersal fish (0.1006 mg/kg WW) followed by in pelagic fish (0.0686 mg/kg WW), freshwater fish (0.045 mg/kg WW), cephalopods (0.0405 mg/kg WW), and crustaceans (0.0356 mg/kg WW). The level of meHg reported in Algerian small pelagic fish (sardine, *Sardina pilchardus*) (0.04 mg/kg WW) is similar to levels from this study. However, in bigger pelagic (swordfish (*Xiphias gladius*); 0.57 mg/kg ww), its level is higher by nearly ten times (Mehouel et al 2019). Similarly, in Taiwan meHg level in swordfish was reported to be five times higher (0.28 mg/kg of meHg) (Hsi et al. 2016). MeHg concentration in other most popular fish consumed by women of childbearing age in Taiwan also showed higher levels compared to this study, except for species like mackerel, milkfish, and anchovy. This group of researchers also reported that meHg levels in tilapia (Hsi et al. 2016) is similar when compared to its level in freshwater catfish captured from this study. Tang et al. (2009, 2014), respectively, reported on relatively low meHg levels in local small-size farmed freshwater or marine whole fish from Hong Kong (0.0045–0.16 µg/g).

In this study, we also calculated fish and seafood consumption pattern by the Malaysian population based on different background which included four different age groups, three major ethnicities, and gender. Data on consumption of higher fish and seafood consumers were also calculated at three centiles of 75th, 90th, and 95th. The main results illustrated the most relevant aspect of fish and seafood consumption patterns for the adolescent and adult population. In our previous fish consumption published data (Ahmad et al 2016), the results were only presented for adults, and discussion has emphasized fish consumption frequencies or most consumed fish and seafood, most preferred cooking style, amounts of fish and seafood consumed by different types and groups, cooking style, and the amount per meal consumed by different ethnics in the country. Published data also described that Malay adolescents in this country consumed seafood most frequently compared to other food groups (Ahmad et al 2019). The consumption data together with levels of meHg in freshwater fish and seafood enables us to calculate and evaluate its contamination status and possible health risk in fish and seafood in Peninsular Malaysia. Table 6 indicated that the risk index (percentage of the PTWI) of meHg is not likely to cause health effects at the estimated mean of fish and seafood consumption at 89 g/day or 623 g/week (median data for overall fish and seafood consumption) using the JECFA PTWI value guideline. The risk index by different demographic factors ranges between 30 and 51% of the PTWI. There were few studies that attempt to compare fish and seafood consumption with dietary intakes estimates of meHg, and the results were similar to this study where the risk index value was lower than the PTWI established by EFSA and JECFA (Mehouel et al 2019; Kuras et al 2017; Tang et al 2009; Tsuchiya et al 2008). Mehouel and co-workers (Mehouel et al. 2019) assessed the risk of meHg intake through consumption of sardine and swordfish fished in three Algerian coasts; the EWI were at 2.8% and 40%, respectively. They highlighted the relationship between trophic levels and biomagnification factors where higher meHg concentration in large predatory fish is due to age, diet, and time of exposure. Intervention research on intake of fish meals based in the Polish subpopulation also revealed the risk index level of up to 38.8% with a range between 22.7 and 59.8%. These researchers also reported the hazard index at 0.39 and revealed that 32.8% of the volunteers exceeded the intake limit by the US-NRC (0.7 µg/kg bw) at 800 g/week of fish consumption (Kuras et al 2017). Another related study is a dietary exposure of Hong Kong secondary school students which showed the estimated exposure to meHg at 25–31% for average fish consumers while the estimation for high consumer was between 75 and 88% of PTWI (Tang et al 2009). Tsuchiya and co-workers (Tsuchiya et al. 2008) conducted a longitudinally study among women of child-bearing age within the Japanese and Korean populations in the state of Washington and reported the differences between levels of total mercury intake by these two populations: the Japanese at 0.09 µg/kg/d or 39% of PTWI and the Korean at 0.05 µg/kg/d or 22% of PTWI.

Our results also revealed that a higher risk index for all demographic groups was contributed from the consumption of marine fish, specifically the demersal group. Despite these results, the highest risk index was shown from the overall consumption of seafood by the adolescent and contributed from the consumption of cephalopod (EWI = 0.8196 µg/kg BW/week). For higher consumers, the EWI values were exceeded the PTWI (risk index > 100%) which also contributed mainly to the consumption of marine fish. The minimum intake of meHg (0.0158 to 0.3488 µg/kg BW/week) was found from the consumption of crustacean in all demographic groups (risk index < 10%) except at a higher rate for the adolescent and Indian ethnicity, which is at 22 and 20%, respectively. We also proved that meHg intake per kilogram body weight depended on species of fish and seafood being consumed. Exposure in some cases was close to the safety margin and observed in top predators and benthic carnivorous fish. Current study reported for this country showed that 14% of seafood had medium to high mercury concentrations with EWI higher than the PTWI. The results appeared for a few species of bream, snapper, croaker, barramundi, and tuna where the EWI values range from 2.1 to 4.0 µg/kg bw (Anual et al 2018). Barone and co-workers (Barone et al. 2015) also reported on this matter where the highest risk index values were calculated from the consumption of such group of fish; the example was from the consumption of European conger eel (1.26 µg/kg bw/week), black belly rosefish (1.22 µg/kg bw/week), long-nose skate (1.12 µg/kg bw/week), swordfish (1.44 µg/kg bw/week), and Atlantic bluefin tuna (1.33 µg/kg bw/week). Although the toxicological evaluation seems to be no important hazard, the levels of meHg in these fishes should be under frequent surveillance. A suggestion has to be made for caution on their consumption by either regular fish consumers or the vulnerable groups of pregnant and lactating women and also young children (Barone et al 2015).

In this current study, we pooled fish and seafood species into a larger group, and the risk index was calculated per group, not for specific species. The present estimations were also consistent with recommended PTWI for the general population, and the emphasis is placed on the toxicity of meHg which essentially accounts from the average data reported by Anual and co-workers (Anual et al. 2018). If we considered the worst-case situation on data for THg in fish and seafood samples, population with all demographic backgrounds would exceed the PTWI defined in the WHO/JECFA guidelines. As it is apparent from these results, a person from a different demographic background can consume 1 to 2 kg of fish and seafood groups weekly, and still the PTWI established by the FAO/WHO will not be exceeded. However, for demersal fish, the same person can only consume < 1 kg (720 g) weekly before exceeding the PTWI value limit of 1.6 μg/kg body weight/week. The health risk exposure associated with the consumption of crustacean analyzed was minor with MSCW of 2 kg and above for all population groups. The exposure diet intake is linked to the HQ which signifies the relationship between the exposure obtained in the diet and the oral reference dose for meHg. The results of this study revealed health risk when HQs were computed for the vulnerable population in the community, while the HQ for adolescents and the elderly reached 1.1227 and 1.0489, respectively. The HQ values close to 1 were associated with the consumption of fish and seafood for adults age > 41 years old, Malay ethnic, both male and female group. Even, HQ for median fish and seafood consumption for the overall population reached 0.8827. The exposure to meHg in this study is likely to exceed the recommended value of PTWI in the case of consumption of high amount of fish and seafood with higher Hg content. The results showed that high consumers consumed fish and seafood at 322 to 406 g/day or 2.3 to 2.8 kg/week. The EWI would be 2.3 to 2.9 µg/kg BW/week or risk index of 146 to 184% compared to the PTWI. These factors caused HQ values to rise above one, ranging from 3.1 to 4.0 for more affluent consumers (fish/seafood intake in the 75th percentile). These results showed that average consumers are doubtful to encounter unnecessary health effects from meHg due to fish and seafood consumption, but the risk is higher for adolescents, the elderly, and high consumers. This study also found that adolescents consumed different types of seafood compared to adults at different demographic factors. They preferred cephalopods and crustaceans, and these groups contributed to the highest mean estimated meHg weekly intakes (EWI) other than from demersal fish. Although the level of meHg in cephalopod and crustacean is lower compared to other marine fish, a high amount of consumption caused these groups of seafood to be significant contributors to meHg accumulation in adolescents in Peninsular Malaysia. Similar patterns of results were reported elsewhere, for example, Andrew et al. (2016) demonstrated the disability-adjusted life years (DALYs) for eating different parts of fish using a risk model (iRISK) and reported the frequency of consumption had exposed the children (< 17 years old) to noncarcinogenic risk, even the amount of Hg in fish part was minute. Their finding also showed that frequent access to tilapia fish in the community and district market attributed to more DALYs. Other findings conducted among secondary school students in Hong Kong revealed that there were no undesirable health effects from consumption of median level of meHg of seafood for both average and high consumers but other sources of meHg, namely shellfish and other seafood products, might add significantly to dietary exposure (Tang et al 2009). Studies on the association between seafood consumption and meHg accumulation revealed a higher average daily dose (ADD) level among the higher seafood consumers who resided in the coastal areas compared to the inland residents (Lee et al. 2012; Jeevanaraj et al 2018).

## Conclusions

The median concentrations of meHg in fish and seafood from Peninsular Malaysia were within the permissible limits by both national and international guidelines. MeHg evaluation seems to be no important hazard associated with average seafood consumers. However, the risk is significantly higher for high consumers when the value of EWI estimated for this group had approached the PTWI. Exposure in some cases was close to the safety margins, thus the meHg level in a certain group of seafood; the demersal fish is recommended to be under frequent surveillance. Regular fish consumers are suggested to be cautious in their consumption of seafood with higher levels of meHg, particularly in young children and the elderly. There is a need for community compassion about risks associated with mercury especially for the vulnerable group. A potential exposure source from consumption of shellfish should be further monitored. There is also a need to investigate the amount of meHg in blood and hair for the total population in the country as human biomonitoring programs are important tools in assessing current population exposure and in discovering trends and patterns related to policies, lifestyle, and food consumption. Information from this study is essential for assessing the effectiveness of policies and advisory authorities in developing relevant consumer recommendations with respect to consumption of seafood and health risk. There is a need to update and refine food consumption databases and levels of meHg in seafood for the purpose of constructing safe-eating guidelines for the public. The limitation of this study is that meHg data in seafood were generated from an earlier study that reported from a limited number of samples. However, this is the nearest estimated which is possible to calculate meHg data for risk assessment estimation to the population in the country.

## Data Availability

The datasets used and/or analyzed during the current study are available from the corresponding author on reasonable request.
